# Attitudes of Croatian pulmonologists concerning obstacles to earlier, more appropriate use of biologics in severe asthma: Survey results

**DOI:** 10.1371/journal.pone.0253468

**Published:** 2021-06-29

**Authors:** Sanja Popović Grle, Marina Lampalo, Sanda Škrinjarić Cincar, Ljiljana Bulat Kardum, Ivan Gudelj, Eugenija Basioli Kasap, Mirna Vergles, Neven Tudorić

**Affiliations:** 1 Clinic for Lung Diseases Jordanovac, University Hospital Center Zagreb, Zagreb, Croatia; 2 School of Medicine, University of Zagreb, Zagreb, Croatia; 3 School of Medicine, University J.J Strossmayer, Osijek, Croatia; 4 Department of Pulmonology, University Hospital Center Osijek, Osijek, Croatia; 5 Section of Pulmonology, Clinic for Internal Medicine, University Hospital Center Rijeka, Rijeka, Croatia; 6 School of Medicine, University of Rijeka, Rijeka, Croatia; 7 Department of Pulmonary Diseases, University Hospital Center Split, Split, Croatia; 8 School of Medicine, University of Split, Split, Croatia; 9 Department of Pulmonology, General Hospital Zadar, Zadar, Croatia; 10 Department of Pulmonology, University Hospital Dubrava, Zagreb, Croatia; Srebrnjak Children’s Hospital, CROATIA

## Abstract

**Aims:**

Biologics have been proven efficacious for patients with severe asthma (SA). It is essential to diagnose such individuals correctly. This study was designed to survey pulmonologists to identify barriers to early diagnosis and subsequent appropriate use of biologics for SA in Croatia.

**Methods:**

A pulmonologist group with expertise in SA developed the initial list of questions, with the final questionnaire created according to a 2-round Delphi method. The resulting survey consisted of 23 items consequently divided into 4 domains: 1) Pulmonologists’ demographics and professional experiences; 2) Concerns about asthma management; 3) Attitudes toward SA diagnosis; and 4) Beliefs and attitudes regarding the use of biologics in managing SA. The given answers represented the respondents’ estimates.

**Results:**

Eighty-four surveys were analyzed, with pulmonologists observing that general practitioners often inaccurately diagnose asthma and treat acute exacerbations. Although specialist centers are capably and correctly equipped, the time to diagnose patients with SA is approximately 3.5 months, with initial use of biologics delayed an additional 2 months. The primary indications for prescribing biologics are conventional therapy with oral glucocorticoids (91.7%) and frequent acute exacerbations (82.1%). In addition to improper diagnosis (64.3%), many patients with SA do not receive the indicated biologics owing to strict administrative directives for reimbursement (70.2%) or limited hospital resources (57.1%).

**Limitations:**

The limitations of this survey include the subjective nature of the collected data, the relatively small sample size, and the lack of the biologic efficacy evaluation.

**Conclusions:**

Croatian pulmonologists observed that a significant number of patients with SA who are eligible for biologics are not prescribed them, largely because of an inaccurate and/or delayed diagnosis, a delayed referral to a specialist center, highly restrictive criteria for reimbursement, and/or institutional budgetary limitations.

## Introduction

Asthma is a heterogeneous disorder characterized by chronic airway inflammation, airway hyper-responsiveness, and variable obstruction that affects more than 358 million people worldwide and is expected to become more and more prevalent with time [1]. Asthma may be effectively treated, with most patients achieving moderate control of the disease. However, 5% to 10% of those with severe asthma (SA), defined as asthma requiring treatment with high-dose inhaled corticosteroids and a second controller and/or systemic corticosteroids or asthma that remains uncontrolled despite such therapy, require additional treatments [2]. Patients with SA are often on maintenance therapy or frequent use of oral glucocorticoids (OCSs) and subsequently exposed to various adverse effects [3]. In individuals with the type 2 (T2)-high endotype of asthma, the biologics targeting IgE (omalizumab), interleukin (IL)-5 (mepolizumab and reslizumab), or IL-5 alpha receptor (IL-5Rα) (benralizumab) significantly improve treatment outcomes [4, 5]. These specified biologic therapies are registered and reimbursed in Croatia, along with strict administrative directives, and can be prescribed only by a pulmonologist in accordance with specific criteria developed by the Croatian Health Insurance Fund (CHIF) and approved by local Pharmacy and Therapeutics (P&T) Committees.

Assessing the precise number of patients with SA in Croatia is difficult because a national registry has not been created. At this point, Croatian patients with SA are registered in the ERS SHARP (European Respiratory Society Severe Heterogeneous Asthma Research Program) registry [6].

The number of patients with SA in Croatia was estimated based on the local epidemiology data for asthma (estimated prevalence of 3%) [7–10] and the Dutch report on SA [11]. In the Dutch study, authors suggested the prevalence of SA in the range of 0.9% (when the most stringent criteria applied: adherence >80%, adequate inhaler use, optimal treatment of contributory factors) and 3.6% of the total asthma population. Applying the quoted estimate, we calculated the range of 1000 to 4000 severe asthmatics in Croatia, half of them being eligible for biologics (500–2000) [12]. However, currently only about 250 adults with SA in Croatia are prescribed biologics, based on personal communications with leaders of regional SA centers. The goal of the study was to identify reasons for the significant discrepancy between SA candidates for biologics and actual SA patients receiving biologics therapy. As the diagnosis and management of SA in Croatia is primarily determined by its pulmonologists, the survey’s aim was to identify the barriers to appropriate prescribing of biologics for SA from the perspective of Croatian pulmonologists.

## Methods

A pulmonologist group with expertise in SA developed the initial list of multiple-choice or fill-in-the-blank questions, with the final survey created according to a 2-round Delphi method [13]. The end-result consisted of a 23-item survey divided into 4 domains: 1) Pulmonologists’ demographics and professional experiences (Q1–Q4); 2) Concerns about asthmatic management (Q5–Q8,Q12), 3) Attitudes toward SA management (Q9–Q11, Q13); 4) Beliefs and attitudes regarding the use of biologics in managing SA (Q14–Q23) (S1 File). The survey was supplemented by the European Respiratory Society/American Thoracic Society definition of SA [2] and Croatian Health Insurance Fund (CHIF)-issued directives for prescribing biologics for SA (S1 Table) and circulated within the network of pulmonologists and residents treating asthmatic patients (through the Croatian Respiratory Society network) and personal contacts of the expert panel members in October 2020. The survey results were evaluated by the authors.

Ethics approval and participant consent were not required; the process of collecting data was anonymous and participating pulmonologists agreed to participate in the study by submitting completed questionnaires.

### Statistical analysis

STATISTICA, version 12 (StatSoft, Inc., www.statsoft.com), was used for statistical analysis. Most questions incorporated descriptive statistics, with the data presented as frequency and percentage or median with interquartile range (IQR), depending on the data. Several answers provided by pulmonologists from university hospitals versus other institutions and between regions were compared using the chi-square test, the Mann-Whitney U Test, and the Kruskal-Wallis ANOVA, with a p value of less than 0.05 considered significant.

## Results

**Fig 1:**
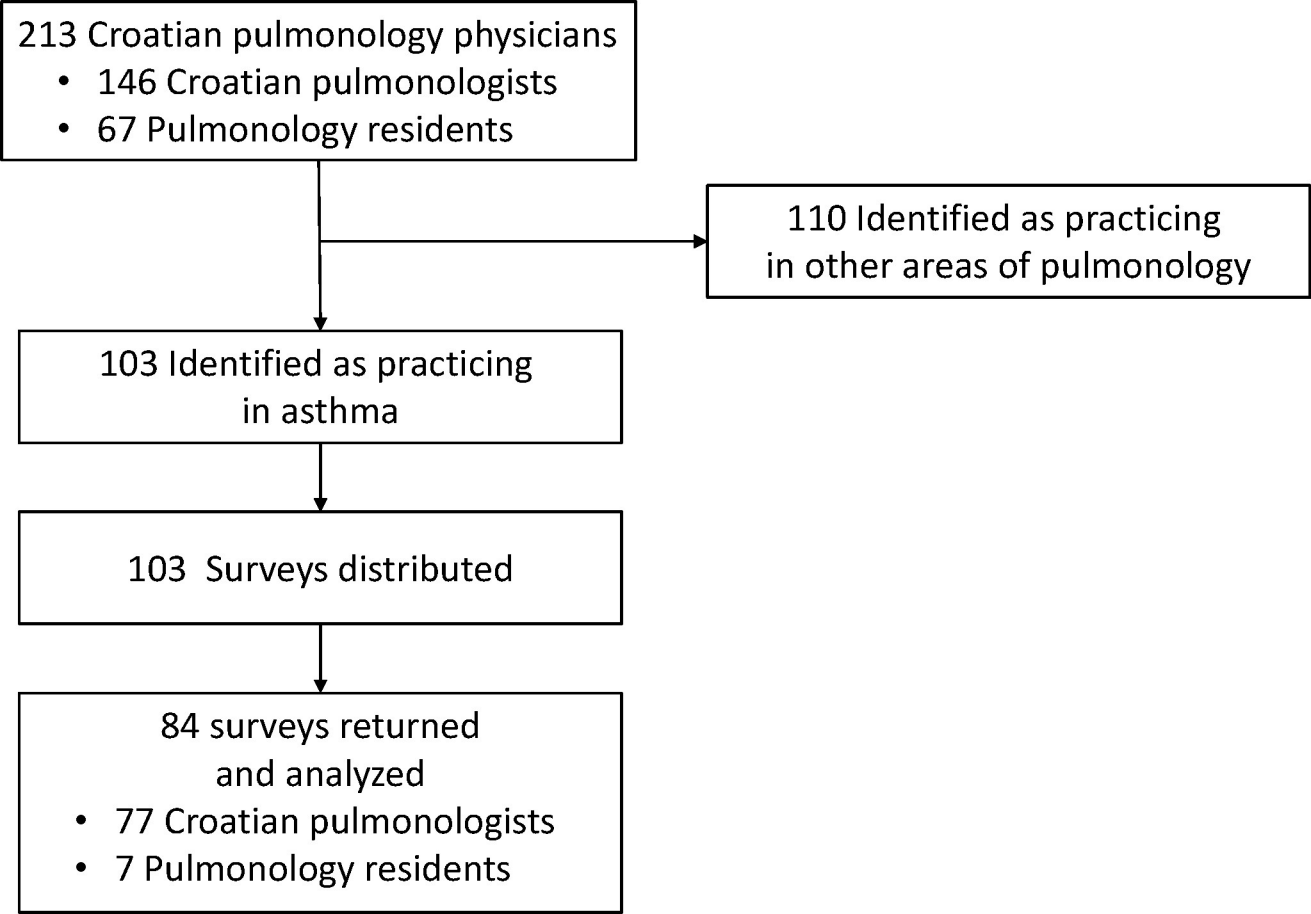
Flow of the participants through the study.

**Fig 2:**
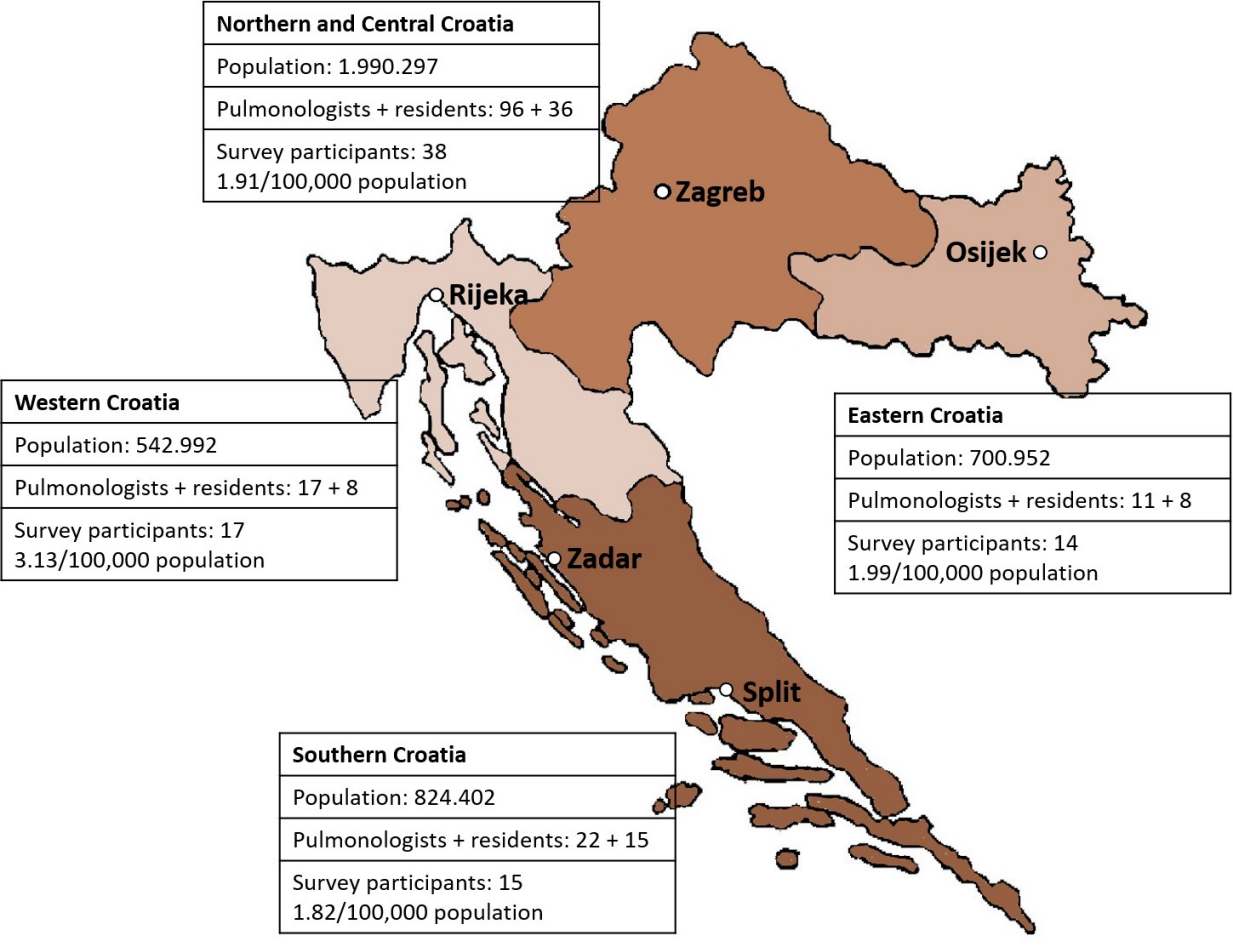
The regional distribution of surveyed pulmonologists. Distribution is shown in relationship to administrative regional division of Croatia into four regions: Northern and Middle, Western, Eastern, and Southern, including data about population, and number of pulmonologists and residents [14, 15]. This also corresponds with location of referral centers for severe asthma in Croatia (two centers in Zagreb, one at Split, Rijeka, Zadar, and Osijek). Reprinted with permission from Kay Square Press, Inc.

A total of 103 surveys [14] were distributed (Fig 1). For the study, Croatia was administratively divided into 4 regions: Northern/Central, Western, Eastern, and Southern, which is conventionally used in the health-care planning [15] and corresponds to the referral center sites for SA in Croatia (two centers in Zagreb, and one in Split, Rijeka, Zadar, and Osijek) (Fig 2).

A total of 84 surveys were completed, returned, and analyzed for this study (Fig 1). More respondents (40.5%) were practicing in university hospitals than in other settings and had more than 10 years of clinical practice in pulmonology (46.4%) (Table 1). More than 80% of respondents treated asthmatic patients daily, with no significant difference between physicians in university hospitals and those in other institutions (chi-square = 1.39, p = 0.5). General practitioners (GPs) more frequently referred their patients to university hospitals compared with other centers (91 vs. 32, z = -3.817, p<0.001, Mann-Whitney U Test). GPs choice to refer their SA patients for treatment did not show regional difference (H = 7.53, p = 0.110, Kruskal-Wallis ANOVA).

**Table 1:** Regional distribution of surveyed pulmonologists (N = 84) and their clinical experiences.

		n	%
Regional centers			
	Osijek	14	16.7
	Split	15	17.8
	Rijeka	17	20.2
	Zagreb	38	45.2
Workplace (Q1)			
	University hospital	34	40.5
	General hospital	29	34.5
	Primary care	5	6.0
	Private practice	4	4.8
	Other^a^	12	14.3
Years in clinical practice (Q2)			
	0–10	39	46.4
	10–20	10	11.9
	20–30	19	22.6
	>30	16	19.0
Experience in managing asthma (Q3)			
	Everyday	68	81.0
	Occasionally	13	15.5
	Only in emergency department	2	2.4
Number of general practitioners referring asthmatic patients (Q4)^b^			
	Median (IQR)	30	20–80

^a^Most (n = 7) are residents, while others are 2 specialists in internal medicine practicing at pulmonology departments, 2 pulmonologists practicing in a rehabilitation hospital, and a pediatrician specialized in asthma.

^b^Data are presented as frequency and percentage or as median and interquartile range (IQR).

Responding pulmonologists widely agreed (90.5%) that GPs rarely initiate diagnostic procedures for asthma, infrequently control the patient’s disease independently (11.9%), and infrequently treat acute exacerbations (23.0%). The time between onset of severe exacerbation and referral to a pulmonologist was seen as unexpectedly long, averaging an estimated 14 days, was similar between regions (p = 0.271), and had no differences between patients who were referred to university hospitals or to other institutions (p = 0.473). The responding pulmonologists also observed that GPs rarely initiate and perform specific diagnostic procedures prior to referral of patients to pulmonologists (Table 2).

**Table 2:** Concerns about clinical issues related to asthmatic management (N = 84).

		N	%
The proportion of GPs beginning initial diagnostics for asthma (Q5)			
	<10%	46	54.8
	10%–30%	30	35.7
	30%–50%	6	7.1
	>50%	2	2.4
Regular follow ups done by (Q6)			
	GP (only prescriptions)	7	8.3
	GP (treatment management)	3	3.6
	GP and pulmonologist	42	50.0
	Pulmonologist	40	47.6
Initial treatment in case of acute exacerbation (%) (Q7)^a^			
	GP during office hours	18	10–30
	GP in a house call visit	5	5–8
	GP in ED	10	9–20
	Specialist in hospital ED	50	30–70
	Pulmonologist in hospital	30	20–50
Average time period from onset of the severe exacerbation until pulmonologist’s visit (Q8)^a^			
	Days	14	7–30
Before referral to pulmonologist, GP indicates following diagnostic procedures (Q12)			
	Spirometry	54	64.3
	Bronchodilator test	11	13.1
	FeNO	3	3.6
	PEFR	8	9.5
	CBC	36	42.9
	Skin prick allergy test	19	22.6
	Chest X-ray	25	29.8

^a^Data are presented as frequency and percentage or as median and interquartile range (IQR).

GP–general practitioner, ED–emergency department, FeNO–fraction of exhaled nitric oxide, PEFR–peak expiratory flow rate, CBC–complete blood count.

The average time required to diagnose patients with SA was 3.5 months (Table 3), with no significant differences noted between university hospitals and other institutions (p = 0.314) or between regions (p = 0.052). Multidisciplinary teams (MDTs) for SA are established primarily in university hospitals compared with other institutions (88.2% vs. 12.2%, chi-square = 47.2, p<0.001). MDTs for SA most commonly included pulmonologists; allergist-immunologists; ear, nose, and throat (ENT) specialists; and psychologists. Nutritionists and physiotherapists were rarely included (<20%). Procedures used for SA phenotyping in more than two-thirds of cases were lung function tests, fraction of exhaled nitric oxide (FeNO) measurement, *in vivo* and *in vitro* allergy tests, complete blood counts, sputum eosinophils, and chest X-ray. These strategies were used significantly more often in university hospitals than in other institutions (11 vs. 9, z = -2.091, p = 0.036).

**Table 3:** Beliefs and attitudes about diagnosing severe asthma (SA) (N = 84).

		N	%
Average time period needed for phenotyping SA (Q9)^a^			
	Days	106	60–180
The existence of a multidisciplinary team for SA (Q10)			
	Yes	36	42.9
Members of the multidisciplinary team for SA in addition to the pulmonologist (Q11) (n = 36)			
	Allergologist/immunologist	25	69.4
	Otorhinolaryngologist	19	52.8
	Nutritionist	1	2.8
	Physiotherapist	7	19.4
	Psychologist	13	36.1
Diagnostic procedures used to phenotype SA (Q13)			
	Spirometry	84	100.0
	Bronchodilator test	84	100.0
	FeNO	74	88.1
	PEFR	60	71.4
	CBC	79	94.0
	Sputum eosinophils	57	67.9
	Skin prick allergy test	78	92.9
	Total and specific IgE	69	82.1
	Bronchial challenge	43	51.2
	Chest X-ray	56	66.7
	HRCT	47	56.0
	Heart US	3	3.6
	CT of paranasal sinuses	6	7.1

^a^Data are presented as frequency and percentage or as median and interquartile range (IQR).

FeNO–fraction of exhaled nitric oxide, PEFR–peak expiratory flow rate, CBC–complete blood count, HRCT–high-resolution computed tomography scan, US–ultrasound.

Respondents’ beliefs and attitudes concerning biologics in SA management (Q14–Q23) are presented in Table 4. ‘Conventional therapy with OCS’ and ‘frequent acute exacerbations’ were the primary indications for prescribing biologics in patients with SA (91.7% and 82.1%, respectively), followed by ‘frequent visits to the emergency department or hospitalizations’ (53.6%). Spirometry reading of severe bronchial obstruction was a more common indication for prescribing biologics in university hospitals than in other institutions (47.1% vs. 20.0%, p = 0.008). The majority of responding physicians recommended biologics in only 1–3 patients, with a small proportion of pulmonologists, mostly members of MDTs, prescribing biologics in most cases. The average time between establishing an indication for biologic therapy and prescribing biologics was approximately 2 months; this interval was significantly shorter in university hospitals than in other institutions (58 days vs. 105 days, z = 2.255, p = 0.024) and showed no regional differences (p = 0.561). Biologics, preferably anti-eosinophil treatments (median 60%), were prescribed primarily in the same institution in which they were prescribed and approved by a P&T Committee. The responding physicians reported that the reasons some patients with SA did not receive the biologics were an improper diagnosis (64.3%), strict administrative directions for reimbursement by the CHIF (70.2%), and/or limited hospital resources (57.1%). This mis-delivery of medication occurred more often in university hospitals than in other institutions (z = -2.626, p = 0.009) and differed significantly between regions (H = 11.73, p = 0.020), with the lowest rate in Eastern Croatia.

**Table 4:** Beliefs and attitudes about biologics in managing severe asthma (SA) (N = 84).

		N	%
Major indications for biologics in SA (Q14)^a^			
	Frequent exacerbations	69	82.1
	Frequent ED visits or hospitalizations	45	53.6
	Severe obstruction present	26	31.0
	Comorbidities	1	1.2
	Maintenance treatment with systemic corticosteroids	77	91.7
	Poor HRQOL	40	47.6
The number of patients for whom you prescribed a biologic in the last 12 months (Q15)			
	0	19	22.6
	1–3	44	52.4
	4–10	16	19.0
	>10	4	4.8
Average time from indication until the actual use of biologic therapy (Q16)^b^			
	Days	60	30–90
Biologic therapy that you indicated was applied as follows (Q17)			
	Not indicated a biologic until now	15	17.9
	In my hospital	47	56.0
	In another hospital	18	21.4
	Was not applied although indicated^c^	2	2.4
Which biologic is, based on phenotyping, most often prescribed in patients with severe asthma in your institution? (Q18)^a^			
	Anti-IgE	30	20–50
	Anti-IL-5 or IL-5R	60	40–70
	Both options	20	10–30
Estimate the number of patients with an established indication for biologics who were not prescribed them because of CHIF directions (Q19)^b^			
	Median (IQR)	5	4–10
How competent are you to diagnose SA? (Q20)			
	Fully competent	31	36.9
	Competent, but lack experience	34	40.5
	Not fully competent due to a lack of experience	16	19.0
	No	3	3.6
Do you consider your institution technically equipped to handle this situation? (Q21)			
	Yes	67	79.8
Indicate the primary reasons as to why patients with SA do not receive biologics even though they should (Q22)			
	SA is not diagnosed by GPs/pulmonologists	54	64.3
	Patients refusing biologic treatment	5	6.0
	Highly strict criteria for reimbursement	59	70.2
	Problems on the level of P&T Committee	16	19.0
	Financial limitations for hospitals/wards	48	57.1
Do you agree that biologics should be available? (Q23)			
	Yes, fully	63	75.0
	Yes, but according to the strict rules of the CHIF	19	22.6
	Yes, if there are funds	3	3.6
	No, other medicines are useful	0	0.0
	No formed viewpoint	0	0.0

^a^3 answers may be chosen.

^b^Data are presented as frequency and percentage or as median and interquartile range (IQR).

^c^Miscommunication and additional diagnostics were the reasons.

ED—emergency department, HRQOL—Health-related quality of life, GPs—general practitioners, P&T Committee–Pharmacy & Therapeutics Committee, CHIF–Croatian Health Insurance Fund.

Significantly more experience and confidence in prescribing biologics was reported from university-hospital pulmonologists compared with other institutions (30.6% vs. 11.8%, chi-square = 9.79, df = 4, p = 0.044). However, nearly 80% of the responding physicians indicated competence in diagnosing and treating SA, while more than half observed that they still required more clinical experience. The same proportion of responding physicians considered their institutions to be adequately equipped to diagnose SA in patients, but they recommended that biologics must be available.

## Discussion

This survey was primarily designed to identify from the perspective of pulmonologists reasons for observed discrepancies in the expected number of candidates for biologic treatments and real-life situations. Other health-care systems with significantly varying health resources share the same challenges. Biologics were prescribed in only 1% of patients with asthma in Bulgaria and fewer than 3 of 1,000 patients with asthma in the United States [16, 17]. In our survey, three primary causes for the disproportionate number of patients receiving biologics for SA were identified: a) an inadequate diagnosis of SA by GPs and pulmonologists and/or referral of patients with uncontrolled asthma to specialist centers; 2) restrictive criteria for the prescription and reimbursement of biologics approved by the CHIF; and c) institutional financial limitations.

Asthma has been frequently reported as under- or overdiagnosed, with both resulting in significant risks to the patient [18–20]. Aaron et al. [19] reported that upon thorough re-examination of 613 Canadian adults diagnosed with asthma, the disorder was ruled out in 33% of patients. In two-thirds of all the cases, asthma had been diagnosed, mostly incorrectly, by family or emergency-department physicians, while in others by a pulmonologist, an allergist, an internist, or a pediatrician. In more than half the patients for whom an asthma diagnosis was ruled out, spirometry or another assessment of variable airflow limitation at the time of asthma diagnosis was not performed [19]. According to pulmonologists’ experiences, our results indicated that Croatian GPs rarely undertake diagnostic procedures to verify asthma quantitatively. In patients with SA, in addition to non-recognition of asthma mimickers, the main barrier to correct diagnosis is poor recognition and comprehension of the severity of the disorder [21, 22]. This lack of appropriate testing and lack of recognition often result in delayed patient referral to specialist care. Based on the results of this survey, appropriate referral to specialist centers of patients with difficult-to-treat asthma or SA is the main challenge in adequate detection and appropriate use of biologics. Our survey results are in line with European expert opinions emphasizing the importance of establishing referral systems and standardizing referral pathways [21, 23]. We also identified a lack of appropriate referrals for patients discharged after an acute exacerbation. The time from onset of the severe exacerbation until the referral to a pulmonologist averaged an estimated 14 days, and was typically related to an unsatisfactory response to the initial treatment given elsewhere. The patient’s improved symptoms, traceable to the acute treatment with systemic corticosteroids, may complicate proper assessment of asthmatic severity. Additionally, the dearth of central registration compiling all acute exacerbations treated at various locations may prevent identification of the most chronic and/or severe patients. Inappropriate referral may well be influenced by GPs who are often unfamiliar with the availability of new medicines. Accordingly, Australian GPs, rarely (21%) refer asthma-exacerbation patients to respiratory specialists, as they consider them to be candidates for treatment with biologic therapy [24, 25].

This survey has shown that Croatian referral centers are capable and correctly equipped for phenotype identification, which is widely accepted as having a major impact on managing SA [21]. Furthermore, the majority of those surveyed expressed confidence in diagnosing SA, although some indicated a need for more experience. This need for more experience was reported most frequently by pulmonologists from non-university hospitals who treated fewer patients with SA and generally made the treatment decisions on their own. Pulmonologists from university centers, however, see more patients with SA and usually rely on a multidisciplinary approach. MDTs located at university hospitals allow for a case management strategy designed to target worsening comorbidities and treatable symptoms [26]. This survey noted that of the few pulmonologists who prescribed biologics frequently, only 5% prescribed these agents in more than 10 patients, exclusively as members of MDTs in referral centers at university hospitals. These findings are in support of the establishment of a network of specialist centers with significant regional distribution. The practice of having many physicians prescribing biologics beyond specialist referral centers has not been shown to be effective [17]. In a recent study, 2358 physicians (56% allergists, 35% pulmonologists, 9% family practitioners) were matched to 4327 prescriptions for a biologic in the treatment of asthma. Nearly two-thirds (65%) of physicians wrote 1 biologic prescription during the study period, frequently with suboptimal patient selection (i.e., individuals with mild disease or non-Th2 endotypes). Many patients had not had an adequate trial of other treatments before receiving biologics for their asthma [17, 27].

Certain barriers even in referral specialist centers may postpone or prevent appropriate use of biologics in SA. Thus, the comprehensive multidisciplinary assessment is often prolonged, as much as an average of 3.5 months in our study, with 25% of cases being delayed >6 months. In the United Kingdom, a requirement for multiple hospital visits to allow use of biologics in SA often postpones the diagnosis several months, costing up to £5,000 per patient [28]. Pulmonologists in our survey commented that the requirement for prescribing biologics, approved by the CHIF, was too restrictive. Namely, the required conditions for all candidates to receive biologics in Croatia (omalizumab, mepolizumab, reslizumab, or benralizumab) are, among other conditions, a forced expiratory volume in 1 second (FEV_1_) of <60% predicted value and at least 4 exacerbations that require the use of OCS (see S1 Table for details).

The primary indications for biologics noted by Croatian pulmonologists in our survey were regular treatment with systemic glucocorticoids and/or frequent exacerbations; severe bronchial obstruction was considered significantly less important. This belief is in line with poor lung function’s not being included in the guidelines as an indication for biologics or was restricted to an FEV_1_ of ≤80% predicted [29, 30]. Albers et al. analyzed the biologic treatment eligibility in a cohort of 502 patients with SA and observed a mean FEV_1_ of 68% of predicted as well as a mean rate of 1.2 significant exacerbations in the previous year [30]. In a *post-hoc* analysis of the same study, approximately 20% of patients eligible for biologics did not reach the requirement, even that of an FEV_1_ of <80%. Furthermore, only 56.9%, 19.0%, 23.9%, and 48.4% patients eligible for mepolizumab, omalizumab, reslizumab, and benralizumab, respectively, had 3 or more exacerbations in the preceding year [31]. Similarly, in a Danish cohort of patients with severe uncontrolled asthma, the mean FEV_1_ was >70%, with only 1 in 10 receiving oral glucocorticoids for more than half of the days in the previous year [32]. This suggests that many patients from these cohorts would not be considered eligible for biologics based on Croatian CHIF criteria.

All responding pulmonologists agreed that biologics should be available for patients diagnosed with uncontrolled SA, with a small proportion of these physicians advocating for the strict requirements of the CHIF. Based on their experiences to date, most participants in this survey believe that anti-interleukin (IL)-5/IL-5Rα biologics are quite effective for most patients with SA, probably owing to the fact that in adult patients with uncontrolled SA, allergy is not the primary pathophysiologic mechanism [29].

Many responding pulmonologists stated that institutional financial limitations are an additional cause for the low rate of approved requests for biologic therapies in some centers. The costs for biologics in Croatia are presently covered by the hospital budgets negotiated and allocated by the CHIF. In some county hospitals, the limited budgets necessitate restrictions on providing high-cost medicines. In such circumstances, biologics for asthma are often not available, usually based on their still unconfirmed cost-effectiveness. In fact, several reports have agreed that despite the demonstrated clinical benefit, reduced exacerbation rates, and improved quality of life of biologics in the treatment of SA, the price would need to be discounted to be considered cost-effective [33–35].

The limitations of this survey include the subjective nature of the collected data and the relatively small sample size. However, the survey included highly competent Croatian pulmonologists from throughout the country who care for patients with SA. Thus, the survey results may assist in appropriate planning measures to improve care for patients with SA in Croatia. This study should also encourage similar surveys in other countries so that appropriate lessons can be learned and improvements made through comparison among asthma services of different countries.

## Conclusions

In conclusion, the responding pulmonologists are aware of the discrepancies between the expected number of candidates seeking biologic treatments and their real-life situations. As the primary causes for these discrepancies, the specialists noted inaccurate and/or delayed diagnosis of SA, slow referral to specialist centers for patients with uncontrolled asthma, excessively restrictive criteria for biologic prescriptions by the CHIF, and institutional budgetary limitations. Therefore, the co-authors concur that many patients with SA are denied the best standard of care. This situation could be substantially improved through earlier identification of patients with difficult-to-treat and SA, by establishing referral systems and standardization of the referral pathways, and by minimizing the time necessary for phenotype identification. In Croatia specifically, omitting the FEV_1_-related requirement for reimbursement of biologics designated by the CHIF would be advantageous.

## Supporting information

S1 File Survey used for the study. (DOCX)

S1 Table National Health Insurance Fund directives for the reimbursement of biologics. (DOCX)
