# Supplementary material for: Attitudes of Croatian pulmonologists concerning obstacles to earlier, more appropriate use of biologics in severe asthma: Survey results
Source: PLoS One. 2021 Jun 29;16(6):e0253468. doi: 10.1371/journal.pone.0253468 (PMC8241034; doi:10.1371/journal.pone.0253468)
Supplement: S1 File — (DOCX) [file pone.0253468.s001.docx]

**Attitudes of Croatian pulmonologists concerning obstacles to earlier, more appropriate use of biologics in severe asthma: survey results**

^1,2^Sanja Popović Grle, MD, PhD; ^1^Marina Lampalo, MD; ^3^Sanda Škrinjarić Cincar, MD, PhD; ^4^Ljiljana Bulat Kardum, MD, PhD; ^5^Ivan Gudelj, MD, PhD; ^6^Eugenija Basioli Kasap, MD; ^7^Mirna Vergles, MD; ^7^Neven Tudorić, MD, PhD.

**Survey used for the study**

| Q1 | Your workplace is: | |  |
| --- | --- | --- | --- |
|  |  | A university hospital | |
|  |  | A general hospital | |
|  |  | Primary care | |
|  |  | Private practice | |
|  |  | Other (name) | |
| Q2 | Number of years practicing as a specialist: | |  |
|  |  | 0—10 | |
|  |  | 10—20 | |
|  |  | 20—30 | |
|  |  | >30 | |
| Q3 | During my daily practice, I work with asthmatic patients: | |  |
|  |  | Every day | |
|  |  | Occasionally | |
|  |  | Only in the emergency department (ED) | |
| Q4 | Estimate the number of general practitioners (GPs) referring asthmatic patients to your institution: | |  |
| Q5 | Estimate the percentage of GPs beginning initial diagnostics for asthma: | |  |
|  |  | <10% | |
|  |  | 10%—30% | |
|  |  | 30%—50% | |
|  |  | >50% | |
| Q6 | Estimate who follows up with patients with diagnosed asthma (you can specify the percentage): | |  |
|  |  | GP (only prescriptions) | |
|  |  | GP (treatment management) | |
|  |  | GP and pulmonologist | |
|  |  | Pulmonologist | |
| Q7 | Initial visit and treatment of patient in the case of acute exacerbation is usually done by: | |  |
|  |  | GP during office hours | |
|  |  | GP in a house call visit | |
|  |  | GP in the ED | |
|  |  | Specialist in the hospital’s ED | |
|  |  | Pulmonologist in the hospital | |
| Q8 | Average time period from onset of severe exacerbation (prescribed systemic corticosteroid) diagnosed in the GP’s office until the referral visit at your institution (specify): | |  |
| Q9 | Average time period required for phenotyping severe asthma (SA) since the appearance of symptoms suggesting severe asthma (specify): | |  |
| Q10 | Do you have a multidisciplinary team for SA in your institution? | |  |
|  |  | Yes | |
|  |  | No | |
| Q11 | Who are the members of the multidisciplinary team for SA? | |  |
|  |  | Pulmonologist | |
|  |  | Allergologist/immunologist | |
|  |  | Otorhinolaryngologist | |
|  |  | Nutritionist | |
|  |  | Physiotherapist | |
|  |  | Psychologist | |
| Q12 | Based on your experience, which diagnostics are indicated by the GP prior to referral to a pulmonologist? | |  |
|  |  | Spirometry | |
|  |  | Bronchodilator test | |
|  |  | FeNO (fraction of exhaled nitric oxide) | |
|  |  | PEFR (peak expiratory flow rate) | |
|  |  | CBC (complete blood count) | |
|  |  | Skin prick allergy test | |
|  |  | Diagnostic imaging (specify): | |
| Q13 | Diagnostic workup used to phenotype SA in your institution: | |  |
|  |  | Spirometry | |
|  |  | Bronchodilator test | |
|  |  | FeNO | |
|  |  | PEFR | |
|  |  | CBC | |
|  |  | Sputum eosinophils | |
|  |  | Skin prick allergy test | |
|  |  | Total and specific IgE | |
|  |  | Bronchial challenge | |
|  |  | Diagnostic imaging (specify): | |
| Q14 | Specify up to 3 major indications for biologics in patients with SA in your practice: | |  |
|  |  | Frequent exacerbations | |
|  |  | Frequent ED visits or hospitalisations | |
|  |  | Severe obstruction on lung function testing | |
|  |  | Comorbidities | |
|  |  | Maintenance treatment with systemic corticosteroids | |
|  |  | Poor health-related quality of life | |
| Q15 | In how many asthmatic patients have you prescribed biologics and requested approval from the Pharmacy & Therapeutics (P&T) Committee or referred patients to another hospital for treatment during the last 12 months? | |  |
|  |  | 0 | |
|  |  | 1—3 | |
|  |  | 4—10 | |
|  |  | >10 | |
| Q16 | Estimate the time period between establishing the indication for biologic therapy until its actual use (specify): | |  |
| Q17 | Biologic therapy that you indicated was applied as follows: | |  |
|  |  | Until now, I have not indicated biologic therapy for SA | |
|  |  | In my hospital | |
|  |  | In another hospital | |
|  |  | Not applied (specify the reason): | |
| Q18 | Which biologic is, based on phenotyping, most often prescribed in patients with severe asthma in your institution? | |  |
|  |  | Anti-IgE | |
|  |  | Anti-interleukin (IL)-5 or IL-5R | |
|  |  | Both options | |
| Q19 | Estimate the number of patients with indications for biologics who were not prescribed them because of CHIF guidelines: | |  |
| Q20 | Are you competent to diagnose SA? | |  |
|  |  | Fully competent | |
|  |  | Feel competent, but lack experience | |
|  |  | Not fully competent due to lack of experience | |
|  |  | No | |
| Q21 | Do you consider your institution technically equipped to diagnose SA? | |  |
|  |  | Yes | |
|  |  | No | |
| Q22 | Indicate up to 3 of the most important reasons that prevent patients with SA from receiving biologics even with indications? | |  |
|  |  | SA is not diagnosed by GPs/pulmonologists | |
|  |  | Patients refusing biologic treatment | |
|  |  | Excessively strict criteria for reimbursement | |
|  |  | Problems at the level of the P&T Committee | |
|  |  | Financial limitations of hospitals/wards | |
| Q23 | Do you agree that biologics should be available for patients with SA? | |  |
|  |  | Yes, fully | |
|  |  | Yes, but according to the CHIF’s strict rules | |
|  |  | Yes, if there is funding | |
|  |  | No, other (existing) medicines are advisable | |
|  |  | No concrete attitude | |
