# Supplementary material for: Attitudes of Croatian pulmonologists concerning obstacles to earlier, more appropriate use of biologics in severe asthma: Survey results
Source: PLoS One. 2021 Jun 29;16(6):e0253468. doi: 10.1371/journal.pone.0253468 (PMC8241034; doi:10.1371/journal.pone.0253468)
Supplement: S1 Table — (DOCX) [file pone.0253468.s002.docx]

S1 Table. National Health Insurance Fund directives for the reimbursement of biologics

| Drug (generic name) | Guideline criteria | | | | |
| --- | --- | --- | --- | --- | --- |
|  | Age (yrs) | Clinical/laboratory | Lung function | Approval | Remarks |
| omalizumab | >6 | - severe persistent IgE-mediated allergic asthma with required continuous or frequent oral corticosteroid therapy (defined as 4 or more cycles during the previous year) - optimal standard therapy (high doses of inhaled corticosteroids, long-acting beta-2 agonists, leukotriene receptor antagonists, theophylline) - positive skin or laboratory test for at least 1 of the inhalation allergens, elevated IgE levels, daily symptoms despite optimal standard therapy | FEV1 <60% | Hospital Medicines Committee | All criteria should be present |
| mepolizumab | >6 | - severe persistent refractory eosinophilic asthma with required continuous or frequent corticosteroid therapy with optimal standard therapy (high doses of inhaled corticosteroids, long-acting beta-2 agonists, leukotriene receptor antagonists, theophylline - GINA severity IV and V) - eosinophils in the peripheral blood >150 cells/μL at the beginning of therapy and >300 cells/μL in the last 12 months - ≥4 exacerbations requiring systemic corticosteroids or continuous treatment with oral corticosteroids at a dosage equivalent to ≥5 mg prednisolone for the past 6 months | FEV1 <60% (for adults)  FEV1 <90% (children and adolescents) | Hospital Medicines Committee | All criteria should be present |
| reslizumab,  benralizumab | >18 | - severe persistent refractory eosinophilic asthma with required continuous or frequent corticosteroid therapy with optimal standard therapy (high doses of inhaled corticosteroids, long-acting beta-2 agonists, leukotriene receptor antagonists, theophylline) - eosinophils in the peripheral blood >150 cells/μL at the beginning of therapy and >300 cells/μL in the past 12 months - ≥4 exacerbations that required the use of systemic corticosteroids or continuous treatment with oral corticosteroids at a dosage equivalent to ≥5 mg prednisolone for the past 6 months | FEV1 <60% | Hospital Medicines Committee | All criteria should be present |
